# Multidimensional assessment of the effects of erenumab in chronic migraine patients with previous unsuccessful preventive treatments: a comprehensive real-world experience

**DOI:** 10.1186/s10194-020-01143-0

**Published:** 2020-06-09

**Authors:** Antonio Russo, Marcello Silvestro, Fabrizio Scotto di Clemente, Francesca Trojsi, Alvino Bisecco, Simona Bonavita, Alessandro Tessitore, Gioacchino Tedeschi

**Affiliations:** 1 Department of Medical, Surgical, Neurological, Metabolic and Aging Sciences, Headache Center, University of Campania “Luigi Vanvitelli”, Piazza Miraglia 2 – I, 80138 Naples, Italy; 2 Institute for Diagnosis and Care, ‘Hermitage-Capodimonte’, Naples, Italy

**Keywords:** Chronic migraine, Erenumab, Migraine, Monoclonal antibodies, Real-world

## Abstract

**Background:**

erenumab was safe and effective in clinical trials for the prevention of migraine. However, real-life data are still lacking. Here we report the clinical experience from an Italian real-world setting using erenumab in patients with chronic migraine experiencing previous unsuccessful preventive treatments.

**Methods:**

Seventy patients with chronic migraine and failure to ≥4 migraine preventive medication classes initially received monthly erenumab 70 mg s.c. Patients without a clinically meaningful improvement, considered as a > 30% reduction in headache days per month, after ≥3 months of therapy switched to monthly erenumab 140 mg. At the first administration and after 3 and 6 months, patients underwent extensive interviews to assess clinical parameters of disease severity and migraine-related disability and impact, and validated questionnaires to explore depression/anxiety, sleep, and quality of life (QoL). Finally, the Pain Catastrophizing Scale, Allodynia Symptom Checklist-12 and MIGraine attacks-Subjective COGnitive impairments scale (MIG-SCOG) were administered.

**Results:**

70% of patients were “responders” after the third administration of erenumab 70 mg, whereas 30% switched to erenumab 140 mg; 29% (6 pts) responded after the sixth administration. The headache-day frequency was reduced from 21.1 ± 0.7 to 11.4 ± 0.9 days after the third administration (*p* < 0.001) and to 8.9 ± 0.7 days after the sixth administration (*p* < 0.001). 53% and 70% of patients, respectively, showed a reduction of ≥50% of headache days/month after the third and the sixth administrations.

Also improved were headache pain severity, migraine-related disability, and impact on daily living, QoL, pain catastrophizing and allodynia (all *p* < 0.001), quality of sleep, symptoms of depression or anxiety (*p* < 0.05) but not MIG-SCOG. There were no new adverse event signals.

**Conclusion:**

These real-world data support monthly erenumab 70 or 140 mg s.c. as a safe and effective preventive treatment to reduce headache frequency and severity in chronic migraine patients experiencing previous unsuccessful preventive treatments.

## Background

The International Classification of Headache Disorders stratifies migraine into episodic or chronic, when patients experience less than or more than 15 days, respectively, of headache per month for at least 3 months, with the migraine features present at least eight days per month [1].

It is of note that chronic migraine, accompanied by functional and microstructural brain abnormalities [2], is associated with a substantially greater personal and societal burden and higher frequency of comorbidities [3]. Nevertheless, although strongly recommended as a crucial component of migraine management, only a minority of patients with chronic migraine (40%) follow preventive therapies and fewer than 25% adhere to preventive medications 1 year after initiating treatment [4], due to low efficacy, side effects or both [3, 5]. Therefore, the standard preventive care based on repositioning drugs discovered by *“serendipity”* still seems to be a challenging issue, particularly with regard to the therapeutic approach to chronic migraine, for which Onabotulinumtoxin type A (onabotulinumtoxinA) is the only guidelines-licensed treatment is [6].

In this context, monoclonal antibodies (mAbs) targeting the calcitonin gene-related peptide (CGRP) or its receptor represent the first selective therapeutic approach specific for migraine prevention [7–12].

Four CGRP antagonists have been developed, undergone experimental testing, and finally been approved as the first selective therapies specifically for migraine prevention: eptinezumab (ALD 403), fremanezumab (TEV-49125), galcanezumab (LY29517542) and erenumab (AMG 334) [7–9]. Eptinezumab, fremanezumab, and galcanezumab bind to the CGRP molecule, whereas erenumab binds to the CGRP receptor [7, 8, 12–14]. Erenumab [15, 16] is a fully human monoclonal antibody shown to be effective and well-tolerated in the preventive therapy of episodic and chronic migraine, with and without medication overuse, even when previous preventive approaches have failed [17].

Recent real-life data from observational studies confirmed that erenumab is highly effective and well-tolerated for the treatment of patients with high-frequency episodic migraine or chronic migraine [18–20]. On the other hand, although the effectiveness of erenumab has been documented in episodic migraine patients with failure of previous preventive treatments and in chronic migraine patients, regardless of previous therapeutic strategies, limited data have been explicitly produced regarding its efficacy in chronic migraine patients who have failed several preventive medication classes [21, 22], which probably represents the most difficult scenario to deal with in clinical practice.

Furthermore, although previous experimental trials showed the safety and effectiveness of erenumab therapy, similar real-life data are still lacking, as well as evidence for its impact on different aspects of the lives of patients.

Herein, we report the clinical experience from an Italian real-world setting using erenumab in a cohort of chronic migraine patients experiencing previous unsuccessful treatments with anti-migraine preventive therapies.

## Methods

### Study design and participants

This was an observational, prospective, non-randomized, open-label study evaluating the efficacy and safety of erenumab in the treatment of chronic migraine patients with failure of previous preventive treatments. Seventy patients with chronic migraine (according to the International Headache Society criteria [1]) were consecutively recruited from the migraine population being referred to the Headache Center of the Department of Neurology at the University of Campania “Luigi Vanvitelli” between February 2019 and July 2019 and followed up for 6 months. We included only chronic migraine patients aged between 18 and 65 years who had received and failed at least four or more oral preventive medication classes (propranolol or metoprolol, topiramate, flunarizine, valproate, amitriptyline, or candesartan) or onabotulinumtoxinA due to lack of efficacy or intolerable side effects. Guided by the exclusion criteria of the available trials and previous real-life studies, migraine patients with concomitant well-defined psychiatric disorders (psychosis, bipolar disorders, or severe depressive symptoms) were excluded from enrolment. The baseline headache frequency (defined as the monthly mean of headaches during the 3 months preceding erenumab treatment) as well as the headache frequency during erenumab treatment were evaluated by reviewing standardized paper patient headache diaries.

Efficacy failure was defined as no meaningful improvement (< 30% of reduction in headache days/month) in the frequency of headaches after the administration of drugs for at least 3 months, as recommended by the European Headache Federation treatment guidelines [23]. Tolerability failure was defined as documented discontinuation due to adverse events at any previous time.

Patients were allowed to take other preventive oral (alone or in combination) or injected therapies if the dose had been stable for at least 3 months before starting treatment with erenumab and remained stable for the entire duration of erenumab treatment.

All patients received monthly erenumab 70 mg subcutaneous (s.c.) until the third administration. Then, patients with a clinically meaningful improvement (≥30% reduction of migraine days per month) [24] continued with the unchanged dose, whereas patients with no clinically meaningful improvement (< 30% reduction of headache days per month) continued with monthly erenumab 140 mg s.c.

At the first administration (T^0^), at the end of the third (T^1^) and of the sixth month (T^2^) of erenumab treatment, all patients underwent an extensive interview aimed at assessing clinical parameters of disease severity such as headache days per month, pain intensity (assessed by numerical rating scale [NRS]), acute pain medication intake and migraine-related disability (by MIDAS) [25, 26] and impact by Headache Impact Test (HIT-6) [27–29]. Furthermore, patients underwent questionnaires aimed at exploring a) comorbid depression and anxiety by the Beck Depression Inventory-II (BDI-II), Hamilton Depression Rating Scale (HDRS) [30], Hamilton Anxiety Rating Scale (HARS) [31]; b) quality of sleep by the Medical Outcomes Study (MOS) Sleep Scale [32] c) quality of life by the migraine-specific quality-of-life questionnaire (MSQ) [33, 34]. Finally, Pain Catastrophizing Scale (PCS) [35], Allodynia Symptom Checklist-12 (ASC-12) [36], and MIGraine attacks - Subjective COGnitive impairments scale (MIG-SCOG) [37] were administered. During the 6 months period of observation, all adverse events (AEs) related to the drug were recorded and used as a safety measure.

The protocol was reviewed and approved by the Ethical Committee of the University of Campania “Luigi Vanvitelli”. Each patient gave a free, informed consent for participation in the study and the analysis and publication of the protocol data. The study was done according to the Strengthening the Reporting of Observational Studies in Epidemiology (STROBE) guidelines [38].

### Outcomes

The primary endpoint was the proportion of patients who achieved at least 30%, 50% and 75% reduction from their individual baseline in monthly headache days at the end of the third (T^1^) and of the sixth month (T^2^) of erenumab treatment. Secondary efficacy endpoints were the change from baseline in monthly headache days, pain intensity, monthly acute migraine medication intake days, and change from baseline in HIT-6, MIDAS, BDI-II, HDRS, HARS, MOS sleep scale, MSQ, ASC-12, MIG-SCOG and PCS scores at the end of the third (T^1^) and of the sixth month (T^2^) of erenumab treatment.

The following secondary endpoints were also assessed: a) percentage of patients converting from medication overuse to non-medication overuse after the third and the sixth monthly erenumab administrations, b) temporal patterns of response to erenumab (e.g., percentage of patients responding in the first month of treatment), c) sustained response to erenumab (e.g., percentage of patients getting a response in the first month and maintaining it in the following 5 months) and d) percentage of non-responder patients at 3 months obtaining a response after a dose increase to 140 mg/month. Finally, a sub-analysis was conducted in those patients previously treated with onabotulinumtoxinA injections.

Safety and tolerability were assessed by recording observed or reported adverse events and by physical examination.

### Statistical analysis

Continuous variables are reported as mean ± standard error (SE), rates and categorical values are reported as subjects-counts and percentage. In the population treated with erenumab, the paired t-test was used to compare the mean headache days, pain intensity, medication intake per month, HIT-6, MIDAS, BDI-II, HDRS, HARS, MOS sleep scale, MSQ, ASC-12, MIG-SCOG and PCS scores at baseline (T^0^) and at the end of the third (T^1^) and of the sixth month (T^2^) of erenumab treatment. Multivariate regression analysis was conducted, including several demographic data (sex, age, migraine onset), and parameters of disease severity (attack frequency, pain intensity, disease duration, MIDAS, HIT-6, ASC-12, PCS scores) to determine the independent predictors of response to erenumab treatment. All analyses were performed using STATA version 14 (StataCorp, College Station, TX, USA).

## Results

### Demographic and baseline headache characteristics

The whole population consisted of 70 patients. The majority of patients were female (78.6%), with a mean age of 46.9 ± 1.4 years (range 18–75). The average time since onset of migraine was 33.1 (± 1.2) years. Demographic and baseline headache characteristics of patients included in the study are reported in Table 1. All patients had experienced multiple failures of preventive treatment, sometimes leading to treatment discontinuation, due to no meaningful improvement (< 30% reduction in headache days per month) or adverse events (Table 2).

**Table 1:** Baseline demographic and clinical parameters

Characteristics	*N* = 70
Age	46.9 ± 1.4
Gender	
Male	15 (21.4)
Female	55 (78.6)
Age at migraine onset, years	14.1 ± 0.9
Disease duration, years	33.1 ± 1.2
Concurrent oral preventive treatments	40 (57)
Monotherapy	18 (26)
Polytherapy	22 (31)
Headache days/month	21.1 ± 0.7
Previous preventive classes failure	4.7 ± 0.3
Acute medications intake/month	25 ± 3.7
Patients with MOH	64 (91.4)
Pain intensity (NRS)	8.6 ± 0.6
MIDAS	108.1 ± 11.4
HIT-6	65.9 ± 1.2
MSQ	13.2 ± 7.5
BDI-II	17.0 ± 1.5
HDRS	14.3 ± 1.2
HARS	17.1 ± 1.7
MOS sleep scale	24.7 ± 0.7
ASC-12	6.7 ± 0.7
PCS	33.2 ± 1.3
MIG-SCOG	9.9 ± 0.6

Values are mean ± standard error (SE) or number (%)

*ASC-12* Allodynia Symptom Checklist-12, *BDI II* Beck Depression Inventory II, *HARS* Hamilton Anxiety Rating Scale, *HDRS* Hamilton Depression Rating Scale, *HIT-6* headache impact test-6, *MIDAS* migraine disability assessment scale, *MIG-SCOG* MIGraine attacks - Subjective COGnitive impairments scale, *MOH* medication overuse headache, *MOS* Medical Outcomes Study, *MSQ* migraine-specific quality-of-life questionnaire, *NRS* numerical rating scale, *PCS* Pain Catastrophizing Scale

**Table 2:** Prior anti-migraine preventive therapies of patients (*N* = 70) in the study, showing pharmacological classes and corresponding reason for failure

	Patients	No meaningful improvement	Adverse events	Treatment discontinuation^a^
Tricyclic antidepressants	65 (93)	55 (85)	10 (15)	48 (68)
Beta-blockers	63 (90)	56 (88)	7 (12)	48 (68)
Calcium channel blockers	28 (40)	22 (78)	6 (12)	25 (36)
Topiramate	62 (88)	47 (76)	15 (24)	50 (71)
Valproate	15 (21)	9 (60)	6 (40)	13 (18)
OnabotulinumtoxinA	54 (77)	54 (100)	–	49 (70)

Values are no. (%)

^a^ Due to no meaningful improvement or adverse event

### Primary efficacy endpoint

**Fig. 1:**
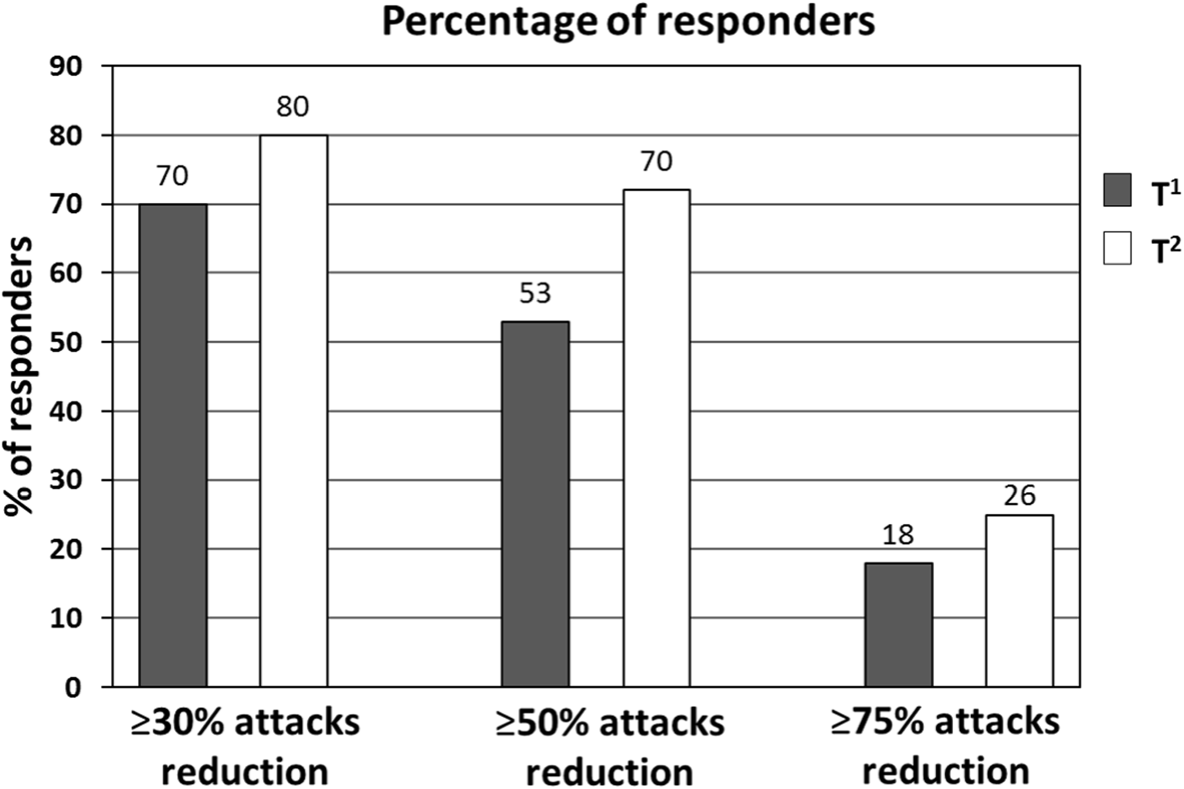
Primary outcome: Percentage of responders at the end of the third (T^1^) and of the sixth month (T^2^) of erenumab administration

Figure 1 shows the percentage of responders at the end of the third (T^1^) and of the sixth month (T^2^) of erenumab administration. After the third administration of monthly erenumab 70 mg s.c., 70% (*n* = 49) of patients were considered “responders” (e.g., ≥30% reduction of headache days/month) and continued with monthly erenumab 70 mg s.c., whereas the remaining 30% (21 pts) were considered “non-responders” (e.g., < 30% reduction of headache days/month) and therefore switched to monthly erenumab 140 mg s.c. Among the latter, 29% (6 pts) were considered “responders” after the subsequent three administrations of monthly erenumab 140 mg s.c.

After the third administration of monthly erenumab 70 mg s.c. 53% (37 pts) and 18% (13 pts) of patients reported respectively a ≥ 50% or a ≥ 75% reduction in headache days per month.

After the sixth administration of monthly erenumab (70 mg s.c. or 140 mg s.c.) 70% (49 pts) and 26% (18 pts) of patients reported respectively a ≥ 50% or a ≥ 75% reduction in the monthly number of headache days when compared to baseline.

### Secondary efficacy endpoints

Statistically significant improvements were observed after the third erenumab 70 mg s.c. administration and then confirmed after the sixth administration (70 mg s.c. or 140 mg s.c.) in the following secondary endpoints (Table 3).

**Table 3:** Efficacy endpoints after the third and sixth monthly erenumab administrations (*n* = 70)

Outcomes	Baseline	Administration	
		Third	Sixth
Reduction from baseline in MHD			
≥ 30%		49 (70)	56 (80)
≥ 50%		37 (53)	49 (70)
≥ 75%		13 (18)	18 (26)
Response after dose increase in non-responder patients	–	–	6/21 (29)
MHD	21.1 ± 0.7	11.4 ± 0.9*	8.9 ± 0.7*
Conversion from chronic to episodic migraine	–	46 (66)	49 (70)
Conversion from medication overuse to non-overuse	–	40 (57)	43 (62)
Pain intensity (NRS)	8.6 ± 0.1	8.1 ± 0.1*	7.9 ± 0.1*
MIDAS	108.1 ± 11.2	54.5 ± 11.4*	51.0 ± 9.7*
HIT-6	65.9 ± 1.2	60.7 ± 1.2*	59.5 ± 1.4*
MSQ	62.7 ± 7.5	42.0 ± 7.6*	41.5 ± 7.7*
BDI-II	17.0 ± 1.4	13.2 ± 1.5	11.2 ± 1.6*
HDRS	14.3 ± 0.9	12.3 ± 1.5	10.5 ± 1.2*
HARS	17.1 ± 1.2	15.1 ± 1.7	13.2 ± 1.6*
PCS	33.2 ± 1.3	24.9 ± 1.8*	25.8 ± 2.1*
MOS Sleep Scale	24.7 ± 0.7	24.2 ± 0.8	22.9 ± 1.1*
ASC-12	6.7 ± 0.7	5.5 ± 0.8*	4.8 ± 0.8*
MIG-SCOG	9.9 ± 0.6	8.6 ± 0.6	8.8 ± 0.8

Values are mean ± standard error (SE) or number (%)

^*^ statistically significant difference (in comparison with baseline)

*ASC-12* Allodynia Symptom Checklist-12, *BDI II* Beck Depression Inventory II, HARS, Hamilton Anxiety Rating Scale, *HDRS* Hamilton Depression Rating Scale, *HIT-6* headache impact test-6, *MIDAS* migraine disability assessment scale, *MIG-SCOG* MIGraine attacks - Subjective COGnitive impairments scale, *MHD* monthly headache days, *MOH* medication overuse headache, *MOS* Medical Outcomes Study, *MSQ* migraine-specific quality-of-life questionnaire, *NRS* numerical rating scale, *PCS* Pain Catastrophizing Scale

**Fig. 2:**
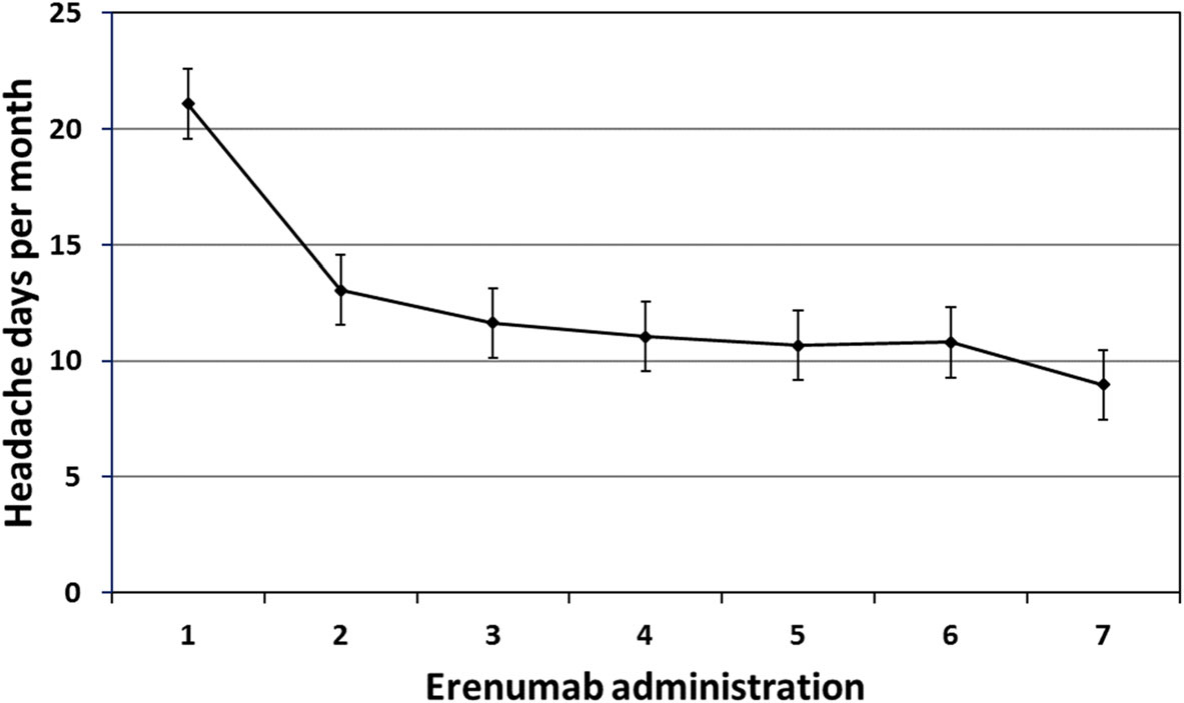
Change in headache-day frequency after each erenumab administration

**Fig. 3:**
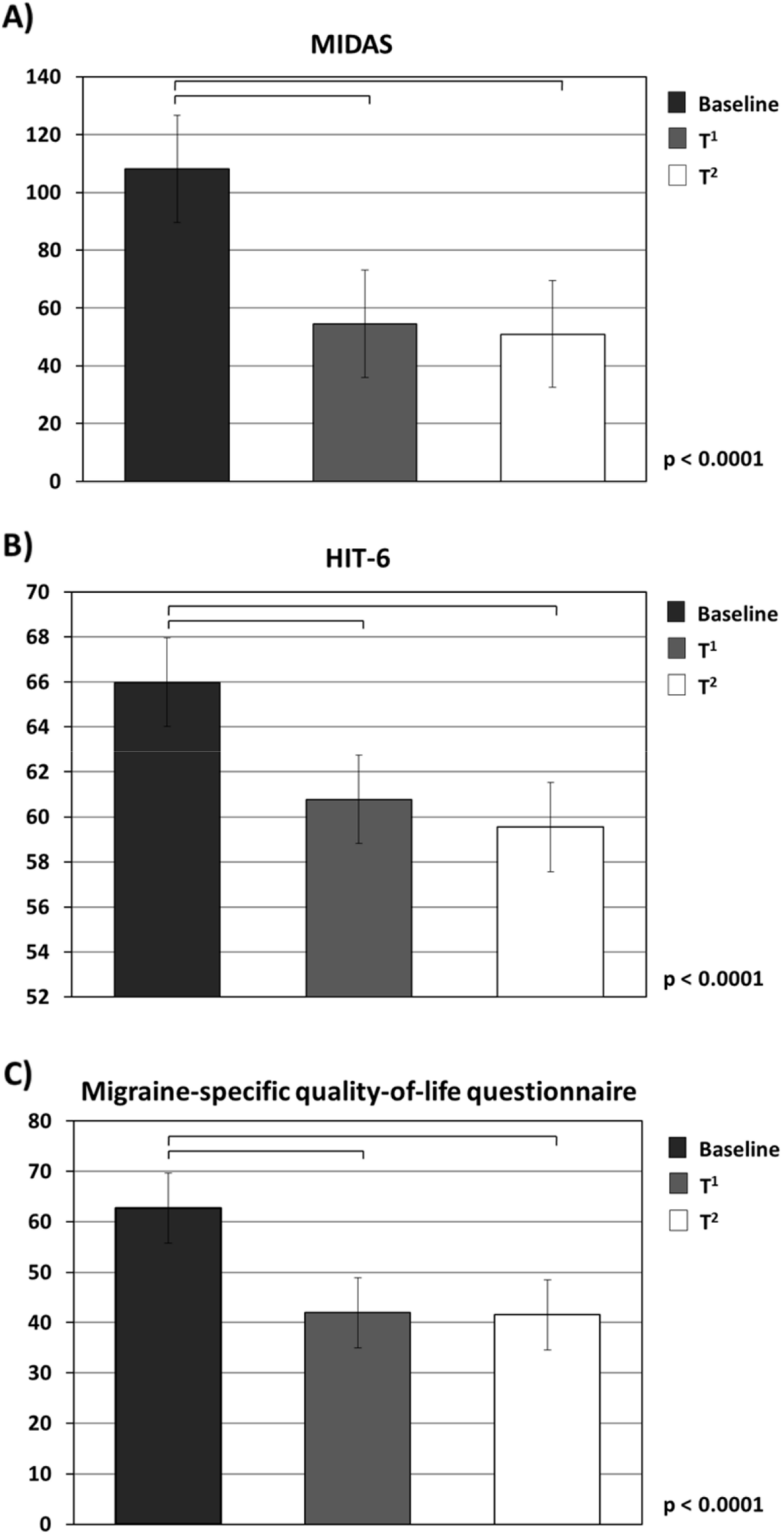
Migraine-related disability and impact on daily living scores at baseline (T^0^), and at the end of the third (T^1^) and of the sixth month (T^2^) of erenumab administration. HIT: headache impact test; MIDAS: migraine-related disability

Headache-day frequency decreased from a baseline mean of 21.1 ± 0.7 to 11.4 ± 0.9 days after the third administration (*p* < 0.001) and to 8.9 ± 0.7 days after the sixth administration (*p* < 0.001) (Fig. 2). Significant improvements compared with baseline were seen in headache pain severity scores assessed by NRS (*p* < 0.001), migraine-related disability assessed by MIDAS and HIT-6 (*p* < 0.001) and impact on daily living assessed by MSQ (*p* < 0.001) after the third and sixth administration (Fig. 3).

**Fig. 4:**
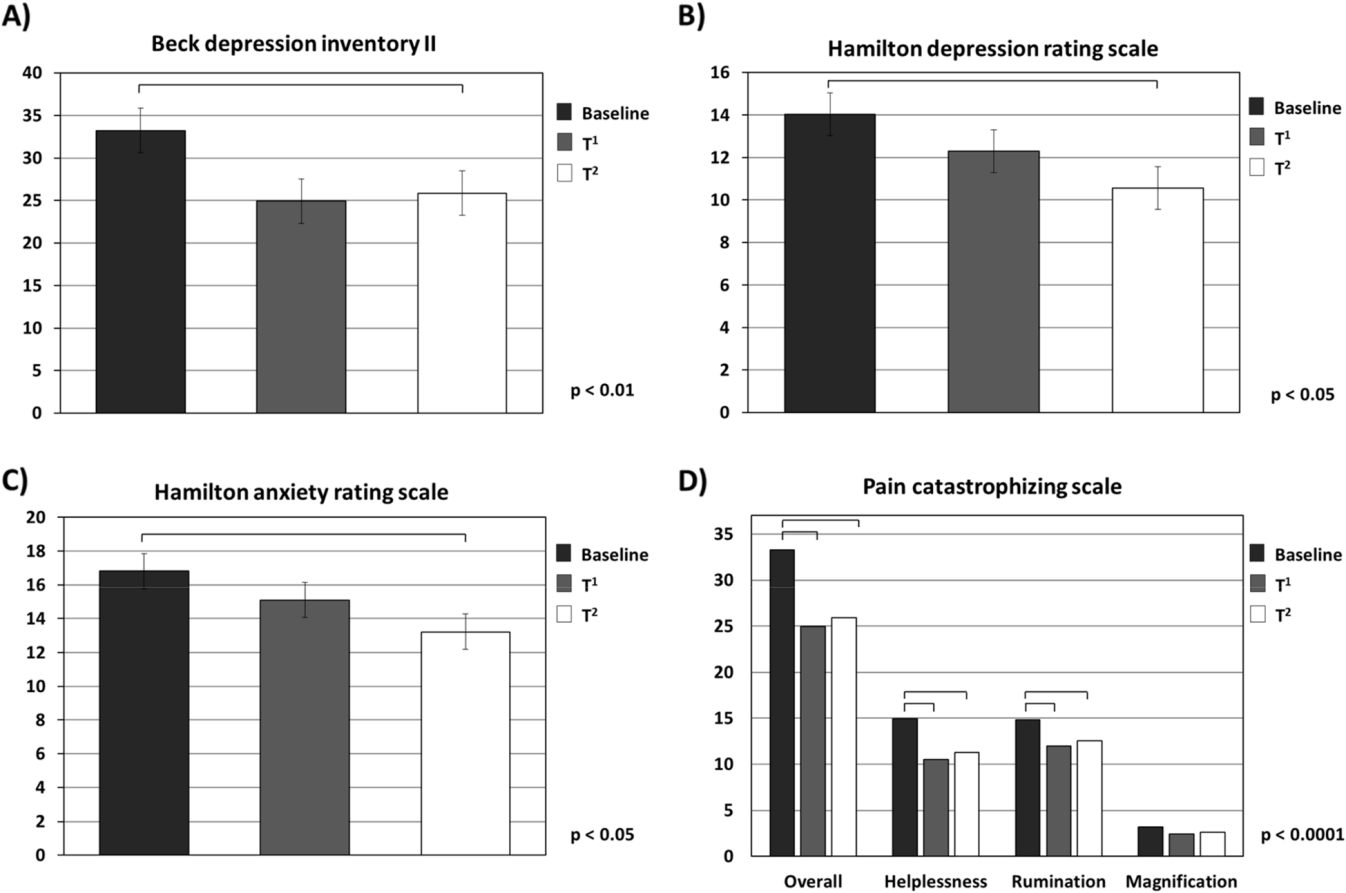
Depressive and anxiety factors and pain catastrophizing scores at baseline (T^0^), and at the end of the third (T^1^) and of the sixth month (T^2^) of erenumab administration

Depression and anxiety assessed by BDI-II, HDRS and HARS (*p* < 0.05) significantly improved from baseline after the sixth administration (Fig. 4a–c), and most measures of pain catastrophizing significantly reduced after the third administration and were maintained throughout treatment (*p* < 0.0001) (Fig. 4d).

**Fig. 5:**
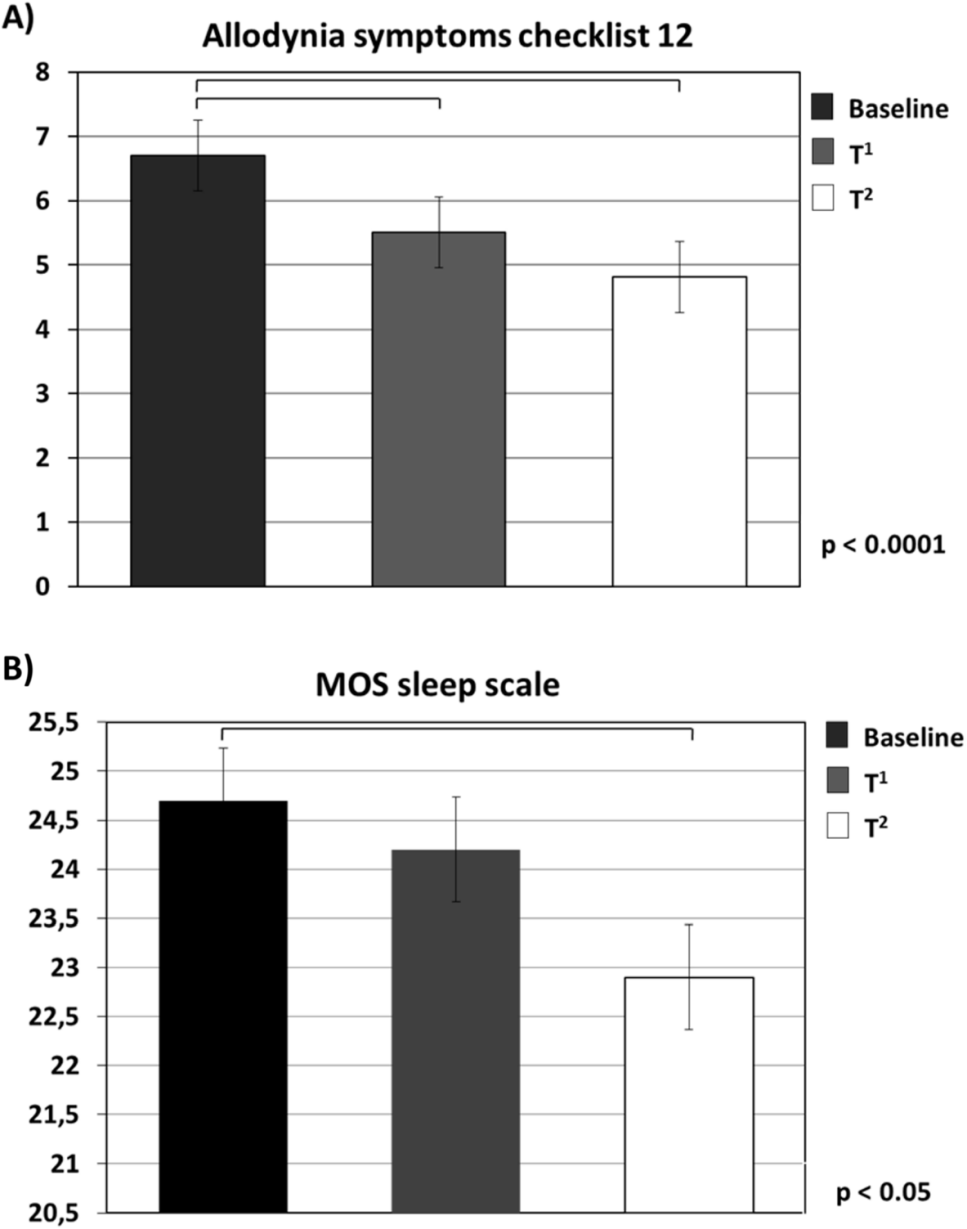
Allodynia Symptom Checklist-12 (ASC-12) scores and Medical Outcomes Study (MOS) sleep scale scores at baseline (T^0^), and at the end of the third (T^1^) and of the sixth month (T^2^) of erenumab administration

Allodynia symptoms assessed by ASC-12 significantly improved after the third and sixth administration (*p* < 0.001) (Fig. 5), and quality of sleep assessed by the MOS sleep scale significantly improved from baseline after the sixth administration (*p* < 0.05) (Fig. 5B).

Subjective cognitive impairment experienced during migraine attacks assessed by MIG-SCOG did not show statistically significant improvement after either the third or sixth administrations.

In the whole cohort of 70 patients, 60% (*n* = 42) showed a ≥ 30% reduction in headache days in the thirty days following the first erenumab administration.

Among the 49 patients considered “responders” after the third administration of monthly erenumab 70 mg s.c, a sustained response after the sixth erenumab administration was observed in 92%. Furthermore, 57% and 62% of patients converted from medication overuse to non-medication overuse after the third and the sixth erenumab administration.

Secondary analysis conducted on 54 patients with a previous failure of onabotulinumtoxinA reported in 56% (30 pts) a ≥ 30% reduction in headache days per month after the sixth erenumab administration.

Analysis of odds ratio (OR) of the multivariate regression analysis found disease duration as a statistically significant negative prognostic factor of response to erenumab treatment (OR: 0.36, *p <* 0.005) (e.g., the shorter the disease duration, the better the erenumab treatment response).

### Safety and tolerability

Treatment-related AEs were consistent with the well-known tolerability profile of erenumab [39]. Overall, 25.7% of patients reported an AE: constipation (23.9%), fatigue (7%), and nausea (5%) were the most frequently reported in the course of treatment. There were no serious AEs, and no patient discontinued treatment due to adverse events.

## Discussion

Chronic migraine imposes a considerable negative burden that affects many important aspects of life, including marital, parenting, romantic and family relationships, career/financial achievement and stability, and overall health [40], and is recognized as the second-highest cause of years lived with disability, and the first in patients aged between 15 and 49 years [41]. Hence, there is a significant unmet need for effective therapies that address the current limited preventive therapeutic options, characterized by frequent unsatisfactory responses, side effects and a consequent poor adherence of patients [3, 42].

The availability of CGRP mABs as novel, well-tolerated, specific, and effective therapeutic options, represents an important advance in the management of migraine prevention [43, 44]. Among CGRP mABS, evidence for the efficacy and safety of erenumab in migraine prevention has been provided from rigorously controlled, randomized, double-blind, placebo-controlled, phase 2 or 3b studies [39, 45–48].

More specifically, a phase 2 study conducted in a cohort of chronic migraine patients showed a reduction of monthly migraine days of 6.6 as compared with 4.2 days in the placebo group [49].

The ARISE study, conducted in episodic migraine patients, showed a reduction of 2.9 days in monthly migraine days, compared with − 1.8 days for placebo and a 50% or greater reduction from baseline in monthly migraine days in the 39.7% of patients treated with monthly erenumab administration compared with 29.5% of the placebo group [50]. Similarly, the STRIVE study showed in a cohort of episodic migraine patients a decrease in monthly migraine days of 3.2 in the 70-mg erenumab group and 3.7 in the 140-mg erenumab group, versus 1.8 days with placebo [51]. Furthermore, a ≥ 50% reduction in the mean number of migraine days per month was reached by 43.3% of patients in the 70-mg erenumab group and 50.0% of patients in the 140-mg erenumab group, as compared with 26.6% in the placebo group. The efficacy and the safety of monthly erenumab administration have also been observed in the LIBERTY study, in a group of episodic migraine patients with failure of two-to-four previous preventive treatments, showing, after the third monthly erenumab administration, at least 50% of reduction in monthly migraine days in the 30% of patients compared with 14% in the placebo group [48]. Altogether, a number of randomized trials have documented the efficacy and safety of erenumab in episodic migraine patients with previous preventive treatment failures and in chronic migraine patients, the latter regardless of previous therapeutic strategies [17, 48, 49].

More recently, data from several real-life studies have confirmed the efficacy and tolerability of erenumab in the treatment of migraine patients with previous preventive failures. Specifically, in a real-life Italian observational study in a cohort of 89 patients with episodic or chronic migraine and at least 2 previous preventive treatment failures, 69.7% of patients had a ≥ 50% improvement in monthly migraine days at or before the third dose of erenumab [19]. Moreover, a German analysis of real-world data from 139 chronic migraine patients who had previously failed both ≥5 previous oral preventive treatments and onabotulinumtoxinA showed a > 30% improvement in monthly headache days in over 50% of patients after receiving at ≥1 injection of erenumab (Table 4) [20].

**Table 4:** Synoptic review of randomized controlled trials and real-life experiences using erenumab as a preventive treatment for migraine

Study	Study design	Type of patients	Preventive medications	Previous treatment failures	Main findings for erenumab compared with controls
Tepper et al. (2017) NCT02066415 [49]	Randomized, double-blind, placebo-controlled, phase 2 trial	Chronic migraine	Not allowed	< 3 (no response)	MMD reduced by 6.6 days vs. 4.2 days with placebo. 40% vs. 41% in the erenumab 70 mg vs. 140 mg groups achieved a > 50% reduction
Dodick et al. (2018) ARISE [50]	Randomized, double-blind, placebo-controlled, phase 3 study	Episodic migraine	Allowed	< 2 (no response)	MMD reduced by 2.9 days vs. 1.8 days with placebo. 39.7% of erenumab recipients achieved ≥50% reduction
Goadsby et al. 2017 STRIVE [51]	Randomized, double-blind, placebo-controlled, phase 3 study	Episodic migraine	Allowed (< 2)	< 2 (no response)	MMD reduced by 3.2 vs. days in the erenumab 70 mg vs. 140 mg group, and by 1.8 days with placebo. 43.3%, 50.0%, and 26.6%, respectively, achieved a ≥ 50% reduction in MMD
Ashina et al. (2018) [52]	Randomized, double-blind, placebo-controlled study with subgroup analyses	Chronic migraine	Not allowed	0, ≥1, ≥2 (no response)	Reduction in MMD vs. placebo for erenumab 70 mg vs. 140 mg: No prior treatment failure, 2.2 vs. 0.5 days; ≥1 prior failed medication category subgroup, 2.5 vs. 3.3 days; ≥2 prior failed medication categories, 2.7 vs. 4.3 days
Reuter et al. (2018) LIBERTY [48]	Randomized, double-blind, placebo-controlled, phase 3b study	Episodic migraine	Not allowed	Failure of 2–4 prior preventive treatments	MMD reduced by > 50% in 30% of patients
Barbanti et al. (2019) [18]	Real-life data	Episodic and chronic migraine	Allowed	Failure of 4–6 prior preventive treatments	≥50% reduction of MMD at weeks 4 and 8, respectively, in 68.2% and 87.5% of chronic patients and 50% and 100% of episodic patients
Ornello et al. (2020) [19]	Real-life data	Episodic and chronic migraine	Allowed	Failure of ≥2 prior preventive treatments	MMD reduced from a mean of 19 days to 4 days. MMD reduced by > 50% in 70% of patients
Raffaelli et al. (2020) [20]	Retrospective real-life	Chronic migraine	Not specified	Failure of 5 prior preventive treatments plus onabotulinumtoxinA	MHD reduction of 3.7 days after the first treatment and 4.7 days after 3 treatment cycles
Robbins et al. (2020) [53]	Retrospective real-life	Chronic migraine	Allowed	Failure of ≥3 prior preventive treatments	MMD reduced by > 30% in 43% of patients

*MHD* monthly headache days, *MMD* monthly migraine days, *vs.* versus

However, no data have been produced prospectively regarding chronic migraine patients, with or without medication overuse, who have previously failed preventive treatments. This is probably the most challenging patient population to deal with in clinical practice. Indeed, to date, only data from a sub-analysis are available in chronic migraine patients who had failed prior preventive treatments, showing reductions in monthly headache days of 2.5, for monthly erenumab 70 mg administration, and 3.3 for monthly erenumab 140 mg administration in patients with ≥ 1 prior failed medication categories, and of 2.7 and 4.3 respectively for monthly administration of erenumab 70 mg and 140 mg in patients with ≥2 prior failed medications [52]. Nevertheless, the above-mentioned erenumab trials did not extensively investigate the impact of erenumab on deeper and overlooked aspects of the migraine burden, such as quality of life, comorbid depressive and anxiety conditions, and sleep quality in patients with chronic migraine.

In the present study, we demonstrated the efficacy and safety of erenumab in a group of 70 chronic migraine patients, with documented failure to at least four migraine preventive medication classes, over a period of 6 months. More specifically, after the third administration of monthly erenumab 70 mg s.c., 70% of patients were considered “responders” (e.g., ≥30% reduction of headache days/month) and continued with monthly erenumab 70 mg s.c., whereas the remaining 30% were considered “non-responders” (e.g., < 30% reduction of headache days/month) and therefore switched to monthly erenumab 140 mg s.c. Among the latter, 29% were considered to have become “responders” after the following three administrations of monthly erenumab 140 mg s.c. After the third administration of monthly erenumab 70 mg s.c. 53% and 18% of patients reported respectively a ≥ 50% or a ≥ 75% reduction in headache days per month. After the sixth administration of monthly erenumab (70 mg s.c. or 140 mg s.c.), 70% and 26% of patients reported respectively a ≥ 50% or a ≥ 75% reduction in the monthly number of headache days compared to baseline.

Although published randomized trials have not shown statistically significant dose-related differences in clinical response between 70 or 140 mg doses of erenumab, our data are in line with sub-analysis of previous randomized trials showing slight clinical advantages of the higher dose in migraine patients with previous preventive treatment failures. We cannot exclude that the higher dosage of erenumab could be necessary to achieve an adequate control of migraine symptoms in patients with a more severe clinical phenotype characterized by failure of previous treatments [54]. Furthermore, to better characterize the benefits of erenumab treatment and to achieve a more comprehensive view of patient experience, beyond the rude counts of headache days or migraine pain intensity scores, several patient-reported outcomes (PROs) were considered. Among these, well-validated questionnaires were employed to evaluate migraine severity, by the HIT-6, to determine how often headaches interfered with activities or caused distress, the MIDAS to assess the number of productive days lost, and the MSQ to measure the effect of migraine on daily functioning. In line with evidence from the literature, a high impact and severe disability, as well as substantial impairments in migraine-specific quality of life, were found in our chronic migraine population. After the third and, even more, after the sixth monthly erenumab administration, a statistically significant improvement across this broad set of PROs became evident. Interestingly, a statistically significant reduction in migraine impact (e.g., by HIT-6) was detected in the 30 days following the first erenumab administration and sustained through the 6-months evaluation.

It is of note that migraine has been widely shown to be associated with depression and anxiety [55]. Although the possibility of common pathophysiological mechanisms is still a matter of debate [56, 57], it is well-known that depressive and anxious conditions make migraine treatment more challenging and are associated with negative outcomes, including increased rates of chronic migraine onset or progression, reduced quality of life and increased overall disease burden [58]. In our population of chronic migraine patients, 75% showed mild depression comorbidity (according to the HDRS or the self-administrated BDI-II), and 58% reported moderate anxiety comorbidity (according to the HARS). A statistically significant improvement in HDRS and self-administered BDI-II scores, achieving values consistent with the absence of depressive contents, and in HARS scores, consistent with the absence of anxiety, were found only after the sixth administration of monthly erenumab, suggesting that longer times are required to improve psychiatric comorbidities in these patients. Interestingly, a remission in depressive symptoms (defined by final BDI-II score < 9) was observed in 23 patients after the sixth administration of monthly erenumab.

Beyond depressive and anxiety factors, migraine patients employ maladaptive pain coping strategies, such as the so-called “pain catastrophizing”, consisting of negative cognitive and affective behavior in response to anticipated or actual pain. In particular, patients who “catastrophize” can experience difficulty in inhibiting thoughts about pain (rumination), exaggerate and worry about the negative consequences of pain (magnification), and believe there is nothing they can do to alleviate the pain (helplessness) [59]. It has been widely demonstrated that pain catastrophizing is associated with increased pain experience and reporting, pain behavior, decreased quality of life, and greater use of healthcare services and longer hospital stays [60, 61]. Among patients with migraine, “catastrophizing” habits are associated with more frequent migraine attacks and chronic migraine, poorer treatment response, increased medical consultation, impaired functioning and reduced health-related quality of life [62]. In this context, the majority of our patients showed PCS scores witnessing negative orientation toward actual or anticipated pain experience. A significant reduction in PCS scores (e.g., mean value below the cut-off value for an aberrant approach to the pain experience) was found after the third monthly erenumab administration, with a better response related to pain rumination and helplessness sub-domains.

Regarding the relationship between sleep and migraine, although the increased incidence and prevalence of sleep disorders in migraine patients is undeniable [63, 64], discussions remain. Insomnia is the most common sleep complaint among migraine patients since it has been observed in 40% of episodic migraine patients and almost 70% of chronic migraine patients, half of which also reporting snoring during sleep [65]. It has been suggested that sleep disorders may predispose individuals to migraine attacks and play a role in migraine chronification [66] and, on the other hand, management of insomnia may reverse chronic in episodic migraine or prevent migraine chronification [67]. Therefore, as sleep disorders could be involved in the onset and resolution of symptoms, they should be carefully considered when discussing migraine management. In the present study, sleep disorders were assessed using the MOS sleep scale, a self-administered scale able to assess 6 different sleep disturbances (difficulty falling asleep and maintaining sleep, daytime sleepiness, respiratory disorders, presence of rhonchopathy, amount of sleep). Global improvement in sleep patterns was demonstrated by a statistically significant reduction of sleep problem index after the sixth monthly erenumab administration.

About two-thirds of migraine patients report cutaneous allodynia, the perception of pain induced by trivial stimuli to normal skin, during or between headache episodes. This is known to represent a risk factor for migraine chronification [68, 69]. In our patient sample, 80% reported ictal cutaneous allodynia at baseline. A statistically significant reduction in ASC-12 values was observed after the third and sixth monthly erenumab administrations, while after the sixth erenumab administration, 13 patients converted to non-allodynia.

Non-pain symptoms, frequently reported by patients with migraine during attacks, could strongly contribute to migraine-related disability [70, 71]. Among these, aside from the “core symptoms” associated with sensory hypersensitivity and neuro-vegetative involvement, cognitive dysfunctions, mainly involving executive and language domains, are often experienced by patients during migraine attacks [72]. Interestingly, in our observation, erenumab did not result in a reduction in the cognitive symptoms associated with migraine attacks, either at the third or sixth monthly erenumab administrations. We speculate that, in acting at the peripheral level of the trigemino-vascular system, longer erenumab treatments could be necessary to re-modulate, even indirectly, the central dysfunctions subtending migraine-related cognitive symptoms.

Finally, our secondary analysis showed that monthly erenumab administration was effective even in a sub-group of patients previously unsuccessfully treated with onabotulinumtoxinA [73].

Multivariate regression analysis illustrated the negative predictive role of disease duration on the response to erenumab treatment. That is, the shorter the disease duration, the better the therapeutic response to erenumab. This finding has also been shown for onabotulinumtoxinA, which is likewise known to work on the CGRP pathway, and could support the early use of erenumab, particularly considering the high efficacy and very good safety profile of erenumab [74, 75].

One of the leading causes of low adherence to preventive migraine treatment is poor tolerability, characterized by systemic and often disabling AEs, such as reduced attention, somnolence, tremor, dizziness, fatigue, depression, loss of appetite, weight gain, hair loss and changes in libido [76]. These side effects were not observed during monthly administrations of either dose of erenumab in the course of 6 months’ observation.

In our study, there were no patient-reported serious AEs or decisions to discontinue treatment due to poor tolerability, although a percentage of migraine patients, higher than those observed by previous erenumab clinical trials, reported constipation, fatigue, and nausea. On the other hand, we cannot exclude that the higher incidence of the AEs reported in our study may reflect an interaction between erenumab and concomitant therapies.

## Conclusion

Our data from the Italian real-world setting support monthly erenumab 70 or 140 mg s.c. as an effective preventive treatment able to reduce headache frequency and severity in a significant percentage of chronic migraine patients experiencing previous unsuccessful preventive treatments. In addition, erenumab showed a significant effect on migraine-related disability and migraine impact on daily living, as well as on both depressive and anxiety symptoms, health-related quality of life, quality of sleep and pain catastrophizing.

This study has many strengths. In particular, although open-label studies with long-term follow-up may be subject to unintentional bias, such as low persistency rates and concomitant medication changes, we observed a persistency of 100% in the absence of changes in preventive medications.

On the other hand, this study is not without limitations. First of all, being a non-randomized open-label study, there was no placebo or active comparator arm. However, an open-label design is informative when the efficacy and safety profile of treatment is established, as it is with erenumab for chronic migraine.

In conclusion, erenumab is not only highly effective but also able to meaningfully alleviate the burden of migraine, with a very low percentage of mild side-effects.

## Data Availability

The data sets analyzed during the current study are available from the corresponding author on reasonable request.

## Abbreviations

AE: Adverse event
ASC: Allodynia Symptom Checklist
BDI-II: Beck Depression Inventory-II
CGRP: Calcitonin gene-related peptide
HARS: Hamilton Anxiety Rating Scale
HDRS: Hamilton Depression Rating Scale
HIT: Headache impact test
mAb: Monoclonal antibody
MIDAS: Migraine-related disability
MIG-SCOG: MIGraine attacks-Subjective COGnitive impairments scale
MOS: Medical Outcomes Study
MSQ: Migraine-specific quality-of-life questionnaire
NRS: Numerical rating scale
PCS: Pain Catastrophizing Scale
QoL: Quality of life
STROBE: Strengthening the Reporting of Observational Studies in Epidemiology
